# Breast cancer epidemiology according to recognized breast cancer risk factors in the Prostate, Lung, Colorectal and Ovarian (PLCO) Cancer Screening Trial Cohort

**DOI:** 10.1186/1471-2407-9-84

**Published:** 2009-03-17

**Authors:** James V Lacey, Aimee R Kreimer, Saundra S Buys, Pamela M Marcus, Shih-Chen Chang, Michael F Leitzmann, Robert N Hoover, Philip C Prorok, Christine D Berg, Patricia Hartge

**Affiliations:** 1 Hormonal and Reproductive Epidemiology Branch, Division of Cancer Epidemiology and Genetics, National Cancer Institute, National Institutes of Health, Rockville, Maryland, USA; 2 Early Detection Research Group, Division of Cancer Prevention, National Institutes of Health, Rockville, Maryland, USA; 3 Infections and Immunoepidemiology Branch, Division of Cancer Epidemiology and Genetics, National Cancer Institute, National Institutes of Health, Rockville, Maryland, USA; 4 Department of Internal Medicine, Huntsman Cancer Institute, University of Utah Health Sciences Center, Salt Lake City, Utah, USA; 5 Biometry Research Group, Division of Cancer Prevention, National Institutes of Health, National Cancer Institute, Rockville Maryland, USA; 6 Global Epidemiology Department, AstraZeneca, Wilmington, Delaware, USA; 7 Nutritional Epidemiology Branch, Division of Cancer Epidemiology and Genetics, National Cancer Institute, National Institutes of Health, Rockville, Maryland, USA; 8 Institute of Epidemiology and Preventive Medicine, University of Regensburg, Regensburg, Germany; 9 Epidemiology and Biostatistics Program, Division of Cancer Epidemiology and Genetics, National Cancer Institute, National Institutes of Health, Rockville Maryland, USA

## Abstract

**Background:**

Multidisciplinary attempts to understand the etiology of breast cancer are expanding to increasingly include new potential markers of disease risk. Those efforts may have maximal scientific and practical influence if new findings are placed in context of the well-understood lifestyle and reproductive risk factors or existing risk prediction models for breast cancer. We therefore evaluated known risk factors for breast cancer in a cancer screening trial that does not have breast cancer as a study endpoint but is large enough to provide numerous analytic opportunities for breast cancer.

**Methods:**

We evaluated risk factors for breast cancer (N = 2085) among 70,575 women who were randomized in the Prostate, Lung, Colorectal and Ovarian Cancer Screening Trial. Using Poisson regression, we calculated adjusted relative risks [RRs, with 95% confidence intervals (CIs)] for lifestyle and reproductive factors during an average of 5 years of follow-up from date of randomization.

**Results:**

As expected, increasing age, nulliparity, positive family history of breast cancer, and use of menopausal hormone therapy were positively associated with breast cancer. Later age at menarche (16 years or older vs. < 12: RR = 0.81, 95% CI, 0.65–1.02) or menopause (55 years or older vs. < 45: RR = 1.29, 95% CI, 1.03–1.62) were less strongly associated with breast cancer than was expected. There were weak positive associations between taller height and heavier weight, and only severe obesity [body mass index (BMI; kg/m^2^) 35 or more vs. 18.5–24.9: RR = 1.21, 95% CI, 1.02–1.43] was statistically significantly associated with breast cancer.

**Conclusion:**

The ongoing PLCO trial offers continued opportunities for new breast cancer investigations, but these analyses suggest that the associations between breast cancer and age at menarche, age at menopause, and obesity might be changing as the underlying demographics of these factors change.

**Clinical Trials Registration:**

http://www.clinicaltrials.gov, NCT00002540.

## Background

Environment, genes, and lifestyle work together to increase or decrease the probability of developing female breast cancer [1]. Events early and late in life consistently influence breast cancer risk [2], but it remains difficult to explain why some women develop breast cancer and others do not [3]. This complicates prevention, yet some groups consistently have notably higher risks than other groups: women whose relatives have breast cancer [4], who first give birth later in life [5], who use exogenous hormones for extended durations [6], and who are overweight or obese after menopause [7].

Most of these conclusions were drawn from individual studies conducted in the 1970s through the late 1990s, or collaborative efforts to quantify risk across many studies [8-10]. Whereas many early studies were exploratory (because risk factor associations were less clear) contemporary studies can have additional or alternative aims, such as searching for molecular markers, evaluating risk among younger women or under-studied racial/ethnic groups, or testing new and refined risk prediction models. Expanding the breast cancer literature this way is obviously necessary, but a logical approach for these studies would be to determine whether the epidemiology of breast cancer in newer studies matches or differs from the published literature to date; because these new foci will likely be most fruitful if placed within the context of known risk factors for breast cancer, both to see whether markers modify those risks or are independently associated with those risk factors [11]. The Prostate, Lung, Colorectal, and Ovarian (PLCO) Cancer Screening Trial [12], although not primarily a breast cancer study, includes extensive data with which to test and develop hypotheses about breast cancer etiology. We therefore analyzed relative risk estimates for etiologic factors linked to breast cancer using the extensive questionnaire data from the approximate 75,000 post-menopausal women in the PLCO study.

## Methods

### Study population

Prorok et al. [13] previously described the full details of the PLCO trial, which includes both men and women. This analysis only includes women. The National Cancer Institute (NCI) designed this trial to determine whether routine screening with chest X-ray, flexible sigmoidoscopy, and cancer antigen 125 (CA-125) plus transvaginal ultrasound could reduce mortality from lung, colorectal, and ovarian cancers, respectively (Clinical Trials Registration: http://www.clinicaltrials.gov, NCT00002540). Recruitment occurred at ten U.S. screening centers (SCs) between 1993 and 2001. Women were considered eligible if they were between ages 55 and 74 and had no previous diagnosis of lung, colorectal, or ovarian cancer. Women who were receiving cancer treatment or participating in another screening or prevention trial were ineligible. Women who had bilateral oophorectomy or were taking tamoxifen were originally ineligible but later allowed to enroll. All participants provided informed consent. Institutional review boards at the NCI and each screening center approved the study. Although the current analysis does not address the PLCO trial's main objectives, the PLCO trial follows the Consolidated Standards of Reporting Trials guidelines [14].

Within-center age- and sex-stratified randomization assigned women to either a control arm, where they were asked to follow their usual medical care, or an intervention arm, where they were offered screening at regular intervals [13]. Participants completed a self-administered risk factor questionnaire at entry, which queried information on demographics, smoking, history of cancer in first-degree relatives, anthropometry, personal medical and medication use history, and personal history of cancer screening tests. Reproductive history covered menarche and menopause, parity and age at first pregnancy, and gynecologic surgeries, including hysterectomy, oophorectomy, and tubal ligation. The questionnaire ascertained age at first use and total duration of use of oral contraceptives. For menopausal hormone therapy, the questionnaire asked about ever-use, current use, and duration of use but did not differentiate estrogen-only formulations from estrogen plus progestin.

### Cancer Ascertainment

Participants received a mailed annual study update (ASU) around each anniversary of their randomization date. The ASUs ascertained the type and date of any cancer diagnosed in the previous year. Study staff contacted non-responding participants by mail and telephone. To validate the self-reported cancers, staff retrieved medical records (for standardized medical record abstraction of pathology reports), death certificates, data from state cancer registries, and information from next-of-kin for deceased participants.

Of 78,231 enrollees (39,116 in the control arm and 39,115 in the intervention arm), 2085 women were diagnosed with breast cancer (including 388 carcinomas in situ) through May 31, 2003. This included 121 (5.8%) self-reported (i.e., unconfirmed) and 1964 (94.2%) confirmed breast cancers. Except where noted, self-reported breast cancers from both arms were included as study endpoints for this analysis; incidence did not differ between arms (data not shown).

### Statistical Analysis

Analysis excluded 659 women (353 from the intervention arm and 306 from the control arm) who never returned an ASU form (and thus for whom breast cancer status was unknown) and women who reported a positive (N = 5119) or unknown (N = 1878) personal history of any cancer before randomization. We considered the 70,575 remaining women at risk for developing breast cancer from the date they completed their baseline questionnaire until the first of the following: breast cancer diagnosis, death, or last ASU.

Follow-up is scheduled for at least 13 years [15]. The mean follow-up time for all women in this analysis was 4.98 years (range, 0.01 years to 9.33 years). The mean (SD) ages at entry and exit were 62.9 years (5.4) and 67.9 years (6.1), respectively. The total number of woman-years of observation was 389,714.

Using Poisson regression in EPICURE software [16], we generated incidence rates and rate ratios (RRs) via standard likelihood ratio methods [17]. Age and calendar time were time-dependent but all other variables were time-independent. We adjusted all RRs for attained age, calendar time, PLCO screening center, age at menarche, age at natural menopause, age at first birth and parity, first-degree family history of breast cancer, benign breast disease, current height, and menopausal hormone therapy use. Complete-case analyses produced similar results (data not shown), so, unless noted, we present analyses that included all participants. We chose the final adjustment factors by comparing models and then dropping potential confounders (e.g., education, marital status, and others) whose presence did not substantially affect the parameter estimates. The final ratio of events to independent variables was approximately 20:1. Randomization produced nearly identical distributions of demographic and reproductive characteristics between arms, and further adjustment for trial arm did not change any results (data not shown).

## Results

Most participants were white but nearly 7,000 (9.7%) were non-white (Table 1). More than 9,000 women (13.7%) reported having a mother, sister, or daughter with a history of breast cancer. More than 5000 women (7.3%) first gave birth after age 30. One third of the participants reported a surgical menopause, approximately one half used oral contraceptives, and two thirds used menopausal hormone therapy. Based on BMI from self-reported height and weight at baseline, one third of the population was overweight (BMI ≥ 25 kg/m^2^) and approximately one quarter was obese (BMI ≥ 30 kg/m^2^) or severely obese (BMI ≥ 35 kg/m^2^).

**Table 1:** Baseline characteristics for 70,575 women in the PLCO Cancer Screening Trial Cohort.

	Number	%
Age at baseline		
55–59	24,381	34.6
60–64	21,405	30.3
65–69	15,405	21.8
70–74	9,384	13.3
Race/ethnicity		
White	62,425	88.5
African-American	4,063	5.8
Asian/Pacific Islander	2,754	3.9
Other*	1,333	1.9
Education		
Less than H.S.	4,598	6.5
Completed H.S.	28,588	40.5
Beyond H.S.	37,215	52.7
Unknown	174	0.3
Marital status		
Married or living as married	48,749	69.1
Widowed	9,700	13.7
Divorced or separated	9,616	12.7
Never married/missing	2,510	4.5
Mother or sister with breast cancer		
No	57,214	81.1
Yes	9,692	13.7
Unknown	3,669	5.2
Age at menarche		
< 12	14,259	20.2
12 – 13	37,816	53.6
14 – 15	15,105	21.4
≥ 16	3,208	4.6
Missing	187	0.3
Years of oral contraceptive use		
None	32,170	45.6
< 1 year	10,219	14.5
2 – 3 years	7,636	10.8
4 – 5 years	5,208	7.4
6 – 9 years	6,303	8.9
≥ 10 years	8,830	12.5
Missing	209	0.3
Parity and age at first birth		
Nulliparous	6,454	9.1
1–2		
< 20 years	2,499	3.5
20–24 years	9,336	13.2
25–29 years	6,629	9.4
30–34 years	2,405	3.4
≥ 35 years	1,021	1.5
3–4		
< 20 years	5,721	8.1
20–24 years	16,227	23.0
25–29 years	5,942	8.4
30–34 years	1,020	1.5
≥ 35 years	343	0.5
≥ 5		
< 20 years	3,818	5.4
20–24 years	7,162	10.2
25–29 years	1,506	2.1
30–34 years	193	0.3
≥ 35 years	194	0.3
Missing or unknown	105	0.2
Menopause Type		
Natural	43,572	61.7
Surgical	23,312	3233.0
Radiation or medications	2,346	3.3
Missing	1345	41.9
Age at Natural Menopause		
< 45 years	4,468	6.3
45–49 years	11,175	15.8
50–54 years	22,134	31.4
55+ years	5,709	8.1
Missing	86	0.1
Menopausal hormone therapy use		
None	23,262	33.0
Former use	11,435	16.2
Current use, ≤ 5 years	11,546	16.4
Current use, > 5 years	23,712	33.6
Missing	620	0.9
Body Mass Index (BMI; kg/m^2^)		
< 18.5	892	1.3
18.5 – 24.9	27,251	38.6
25 – 29	24,214	34.3
30 – 34.9	11,102	15.7
≥ 35	6,297	8.9
Missing	819	1.2

*Other race/ethnicity includes Hispanic, American Indian, and missing/unknown.

As expected, age-specific breast cancer incidence rates rose with increasing age (Table 2). Compared with white women, African-American and Asian/Pacific Islander women were non-significantly more likely to develop breast cancer after adjustment for other factors (see table footnote). Increasing parity and earlier age at first birth were inversely associated with breast cancer. Later ages at menarche and natural menopause were inversely and positively, respectively, associated with breast cancer. Current menopausal hormone therapy use at baseline, regardless of duration, significantly increased risk, but the baseline questionnaire did not query hormone therapy formulation or regimen. Height, weight, and BMI were positively associated with breast cancer, although weight and BMI associations emerged only after statistical adjustment and only a few categories produced statistically significant associations.

**Table 2:** Number of breast cancers, person-years, rates, and RRs by demographic, reproductive, and anthropometric factors.

	Breast Cancers*	Woman-Years	Crude Rate	RR**	95% CI
Attained Age					
55–59	274	63,088	434.31	1.00	Ref.
60–64	608	108,640	559.65	1.36	1.18–1.57
65–69	582	104,433	557.30	1.44	1.24–1.67
70–74	434	79,134	548.44	1.48	1.26–1.73
75+	187	34,419	543.30	1.47	1.21–1.79
Race					
White	1,854	345,926	535.95	1.00	Ref.
African-American	89	19,765	450.29	1.05	0.84–1.32
Asian/Pacific Islander	111	17,230	644.23	1.14	0.86–1.51
Other/unknown	31	6,793	456.35	0.92	0.64–1.32
Family history of breast cancer					
No	1696	336,410	504.15	1.00	Ref.
Yes	389	53,304	729.78	1.44	1.29–1.60
Number of Live Births					
0	240	35,545	675.20	1.00	Ref.
1	160	28,296	565.45	0.70	0.55–0.89
2	524	88,510	592.02	0.76	0.62–0.92
3	526	96,163	546.99	0.75	0.62–0.91
4	331	66,673	496.45	0.72	0.59–0.88
5+	301	73,888	407.37	0.65	0.53–0.80
Age at First Birth					
None	240	35,545	675.20	1.00	Ref.
< 20	267	62,165	429.50	0.68	0.53–0.86
20–24	901	181,709	495.85	0.74	0.59–0.91
25–29	471	80,523	584.93	0.83	0.66–1.03
30–34	148	20,822	710.79	1.03	0.81–1.31
35+	47	6,894	681.75	1.02	0.74–1.41
Age at Menarche					
< 12 years	457	76,987	593.61	1.00	Ref.
12–13 years	1099	209,082	525.63	0.86	0.77–0.97
14–15 years	439	84,595	518.94	0.85	0.74–0.97
≥ 16 years	88	17,874	492.34	0.81	0.65–1.02
Age at Menopause					
< 45 years	123	25,254	487.05	1.00	Ref.
45–49 years	340	64,113	530.31	1.07	0.87–1.31
50–54 years	700	126,080	555.20	1.12	0.92–1.35
55+ years	211	32,192	655.44	1.29	1.03–1.62
Surgical menopause	574	122,386	469.01	0.84	0.69–1.02
Radiation/medication	94	12,061	779.37	1.32	1.01–1.74
Menopausal hormone therapy use					
Never	571	134,329	425.08	1.00	Ref.
Former	280	64,773	432.28	1.02	0.88–1.18
Current, < 5 years	372	61,931	600.67	1.44	1.26–1.66
Current, ≥ 5 years	847	125,006	677.57	1.67	1.49–1.87
Weight (kg)					
< 60	437	82,961	526.75	1.00	Ref.
60 – 64.9	310	59,068	524.82	1.03	0.86–1.19
65 – 69.9	311	56,091	554.46	1.18	0.97–1.30
70 – 74.9	257	49,325	521.03	1.08	0.92–1.26
75 – 79.9	267	47,072	567.22	1.21	1.03–1.42
≥ 80	486	91,892	528.88	1.20	1.04–1.38
Height (m)					
< 1.60	527	101,460	519.42	1.00	Ref.
1.60 – 1.64	605	117,236	516.05	1.01	0.89–1.13
1.65 – 1.69	561	102,205	548.90	1.06	0.94–1.20
≥ 1.70	385	66,707	577.15	1.11	0.97–1.27
BMI (kg/m^2^)					
< 18.5	25	5,135	486.85	0.88	0.59–1.32
18.5 – 24.9	848	154,447	549.06	1.00	Ref.
25 – 29	712	133,799	532.14	1.06	0.95–1.17
30 – 34.9	305	59,109	516.00	1.10	0.97–1.26
≥ 35	173	32,478	532.67	1.21	1.02–1.43

* Includes confirmed invasive cancers, confirmed in situ lesions, and self-reported cancers.

** Adjusted for attained age, screening center, age at menarche, age at menopause, family history of breast cancer, benign breast disease, height, and menopausal hormone therapy (all in categories as shown in the table), plus combined age at first birth and parity (nulliparous or first birth after 35 years, first birth before age 25, first birth 25–34 years, or unknown) and calendar time (5/31/1993 – 5/31/1995, 6/1/1995 – 5/31/1997, 6/1/1997 – 5/31/1999, 6/1/1999 – 5/31/2001, and 6/1/2001 – 5/31/2003). RRs for age at first birth and parity were adjusted for the other factor.

Not shown are unknown parity (3 breast cancers and 638 person-years), age at first birth (11 breast cancers and 2,635 person-years), age at menarche (2 breast cancers and 1,177 person-years), age at or type of menopause (43 breast cancers and 7,628 person-years), menopausal hormone therapy use (15 breast cancers and 3,676 person-years), weight (17 breast cancers and 3,304 person-years), height (7 breast cancers and 2,107 person-years), and BMI (22 breast cancers and 4,747 person-years).

## Discussion

Our analysis of postmenopausal breast cancer in the PLCO study revealed interesting differences from the generally established epidemiology of breast cancer. Increasing age, parity, family history of breast cancer, and use of menopausal hormone therapy were all associated with breast cancer as expected. Associations with other key risk factors – age at menarche, age at menopause, and obesity – were slightly different from the previously published literature. These differences have at least two implications for ongoing and future research on breast cancer.

First, the magnitude of some risk factor associations may be changing. Published studies have reported consistent, linear risk relationships with older ages at menarche (e.g.: 5% decrease in risk per 1-year delay after age 12) and menopause (e.g.: 3% increase in risk per 1-year delay age at menopause)[8,9,18]. In contrast, we observed a fairly uniform decreased risk with older ages at menarche and a non-significant increased risk only in the oldest age-at-menopause group. The larger numbers of older women in the PLCO cohort could explain the lower RRs. Alternatively, inaccurate recall, especially among older women [19], or non-differential misclassification could be a factor, because our questionnaire collected only categorical data on these ages.

Body size is positively associated with postmenopausal breast cancer [20]. In a pooled analysis of cohort studies, risk increased significantly by 7% per 4-kg/m^2 ^BMI increase, 7% per 5-cm height increase, and 6% per 10-kg weight increase [7]. In our analysis, all three factors increased the relative risks by 20%. Despite the large sample size, only the RRs for the highest categories were statistically significant; the BMI association was stronger than the height and weight associations.

We observed a higher RR for breast cancer for the combination of low parity and later age at first birth, whereas an earlier meta-analysis reported declining RRs with lower parity in women whose first birth occurred at older ages [21]. Both that and our study included few multiparous women who first gave birth after age 35.

These changes could reflect underlying demographic changes. Mean age of menarche among U.S. females has declined in recent decades, whereas later age at natural menopause is more common than before [22,23]. The prevalence of women who first give birth after age 35 is increasing, as is the prevalence of obesity [24]. These factors are potentially related: obesity might spark early estrogen production and the onset of puberty, whereas parity and BMI are also associated with later age at menopause [25-28]. Teasing apart the quantitative effect of these changes on risk factor associations, as well as the underlying biologic implications, may prove to be a challenge.

Changing distributions of risk factors will affect the use of risk prediction models that project individual probabilities of breast cancer and influence eligibility for clinical trials. The widely used Gail model [29] relies on readily available medical information, such as age at first birth and age at menopause. Modified prediction models incorporate additional clinical information, such as breast density [30]. If the relative risk measures that underlie the projection of absolute risks in these models are changing, then there is the potential for the models to lose some of their current calibratory and discriminatory ability. Continued assessment of model performance among newer population-based studies with diverse populations could address this and shed further light on the potential influence of changing demographics on the epidemiology of postmenopausal breast cancer.

The known risk factors for breast cancer account for perhaps only 50% of the population burden of breast cancer [31]. A polygenic model of breast cancer hypothesizes that many genetic factors contribute individually small but collectively large effects that could explain the remaining 50% of the population attributable risk [32]. Based on extensive results to date of candidate pathways, the overall effect of low-penetrance SNPs is minimal [33]. The SNP-based associations that have emerged from marker-based scans have unknown function or functions unrelated to the hormonal pathways linked with breast cancer [34,35]. Other important genetic markers with relevant functions might surface. Exploration of genetic factors is likely to be most fruitful if placed within the context of the known risk factors for breast cancer, both to see whether markers modify those risks or are independently associated with those risk factors [11].

Whether known or future genetic markers can improve the performance of existing risk prediction models – or potential new models that incorporate the clinical heterogeneity of breast cancers [36]-is uncertain. Readily available lifestyle or questionnaire-based information, such as reproductive history, will remain the cornerstone of risk prediction and stratification even if it becomes easy, inexpensive, and risk-free for large numbers of women to determine their genetic profile because the small-magnitude risk associations are unlikely to be useful for prediction [37,38]. Changes in the underlying associations between those risk factors and breast cancer would not adversely affect such evaluation, but it would require continued surveillance of breast cancer epidemiology among contemporary populations, such as PLCO, both individually and within large-scale replication efforts [32].

Our large sample size makes it unlikely that the deviations from expectation that we observed were due to chance. Overall exposure and endpoint data were likely good, but residual confounding might exist. The questionnaire lacked information on some risk factors, such as breastfeeding [8], physical activity [39], and alcohol use [40]. For others – particularly menopausal hormone therapy [41] – the baseline questionnaire did not allow us to differentiate the higher-risk estrogen-plus-progestin formulations from estrogen-only formulations. Compared with 2001 U.S. Census Bureau data on women aged 55–74 [42], lower percentages of PLCO participants reported receiving some formal education beyond high school across all racial/ethnic groups: 58% vs. 53% for whites, 67% vs. 52% for African-Americans, and 57% vs. 52% for Asian/Pacific Islanders. We cannot rule out that other unmeasured factors may make our study population slightly different from the U.S. population on the whole. Our analysis covered a relatively short follow-up, and continued follow-up of the PLCO population may confirm both the validity and generalizability of our findings.

## Conclusion

In conclusion, this study of over 75,000 post-menopausal women suggests that population-level and demographic changes might influence the magnitude of well-established associations between certain recognized risk factors and breast cancer. These potential changes may become increasingly important as new molecular epidemiologic efforts attempt to expand upon existing methods for understanding relative and absolute risks for breast cancer.

## Competing interests

The authors declare that they have no competing interests.

## Authors' contributions

JVL and PH designed the investigation. JVL analyzed the data. JVL, ARK, SSB, PMM, SCC, MFL, RNH, PCP, CDB and PH interpreted the results. JVL, ARK, SSB, CDB, and PD drafted the manuscript, and JVL, ARK, SSB, PMM, SCC, MFL, RNH, PCP, CDB, and PH edited the manuscript.

## Pre-publication history

The pre-publication history for this paper can be accessed here:

http://www.biomedcentral.com/1471-2407/9/84/prepub

## References

[B1] Hartge P Genes, hormones, and pathways to breast cancer N Engl J Med 2003 348 2352 2354 10.1056/NEJMe030065 12789001

[B2] Hankinson SE Colditz GA Willett WC Towards an integrated model for breast cancer etiology: the lifelong interplay of genes, lifestyle, and hormones Breast Cancer Res 2004 6 213 218 15318928 10.1186/bcr921 PMC549181

[B3] Rockhill B Weinberg CR Newman B Population attributable fraction estimation for established breast cancer risk factors: considering the issues of high prevalence and unmodifiability Am J Epidemiol 1998 147 826 833 9583712 10.1093/oxfordjournals.aje.a009535

[B4] Familial breast cancer: collaborative reanalysis of individual data from 52 epidemiological studies including 58,209 women with breast cancer and 101,986 women without the disease Lancet 2001 358 1389 1399 10.1016/S0140-6736(01)06524-2 11705483

[B5] Albrektsen G Heuch I Hansen S Kvale G Breast cancer risk by age at birth, time since birth and time intervals between births: exploring interaction effects Br J Cancer 2005 92 167 175 10.1038/sj.bjc.6602302 15597097 PMC2361726

[B6] Beral V Breast cancer and hormone-replacement therapy in the Million Women Study Lancet 2003 362 419 427 10.1016/S0140-6736(03)14596-5 12927427

[B7] Brandt PA van den Spiegelman D Yaun SS Adami HO Beeson L Folsom AR Fraser G Goldbohm RA Graham S Kushi L Pooled analysis of prospective cohort studies on height, weight, and breast cancer risk Am J Epidemiol 2000 152 514 527 10.1093/aje/152.6.514 10997541

[B8] Collaborative Group on Hormonal Factors in Breast Cancer Breast cancer and breastfeeding: collaborative reanalysis of individual data from 47 epidemiological studies in 30 countries, including 50302 women with breast cancer and 96973 women without the disease Lancet 2002 360 9328 187 195 12133652 10.1016/S0140-6736(02)09454-0

[B9] Hunter DJ Spiegelman D Adami HO Brandt PA van den Folsom AR Goldbohm RA Graham S Howe GR Kushi LH Marshall JR Non-dietary factors as risk factors for breast cancer, and as effect modifiers of the association of fat intake and risk of breast cancer Cancer Causes Control 1997 8 49 56 10.1023/A:1018431104786 9051322

[B10] Key T Appleby P Barnes I Reeves G Endogenous sex hormones and breast cancer in postmenopausal women: reanalysis of nine prospective studies J Natl Cancer Inst 2002 94 606 616 11959894 10.1093/jnci/94.8.606

[B11] Chanock SJ Hunter DJ Genomics: when the smoke clears Nature 2008 452 537 538 10.1038/452537a 18385720

[B12] Hayes RB Reding D Kopp W Subar AF Bhat N Rothman N Caporaso N Ziegler RG Johnson CC Weissfeld JL Etiologic and early marker studies in the prostate, lung, colorectal and ovarian (PLCO) cancer screening trial Control Clin Trials 2000 21 349S 355S 10.1016/S0197-2456(00)00101-X 11189687

[B13] Prorok PC Andriole GL Bresalier RS Buys SS Chia D Crawford ED Fogel R Gelmann EP Gilbert F Hasson MA Design of the Prostate, Lung, Colorectal and Ovarian (PLCO) Cancer Screening Trial Control Clin Trials 2000 21 273S 309S 10.1016/S0197-2456(00)00098-2 11189684

[B14] Moher D Schulz KF Altman D The CONSORT statement: revised recommendations for improving the quality of reports of parallel-group randomized trials JAMA 2001 285 1987 1991 10.1001/jama.285.15.1987 11308435

[B15] Gohagan JK Prorok PC Hayes RB Kramer BS The Prostate, Lung, Colorectal and Ovarian (PLCO) Cancer Screening Trial of the National Cancer Institute: history, organization, and status Control Clin Trials 2000 21 251S 272S 10.1016/S0197-2456(00)00097-0 11189683

[B16] Preston DL Lubin J Pierce DA McConney ME EPICURE [software] 1996 Seattle, WA: HiroSoft International Corp

[B17] Breslow NE Day NE Statistical methods in cancer research. The design and analysis of cohort studies 1987 II Lyon: International Agency for Research on Cancer 3329634

[B18] Collaborative Group on Hormonal Factors in Breast Cancer Breast cancer and hormone replacement therapy: collaborative reanalysis of data from from 51 epidemiological studies of 52,705 women with breast cancer and 108,411 women without breast cancer Lancet 1997 350 1047 1059 Erratum in: Lancet 1997 15;350(9089):1484. 10.1016/S0140-6736(97)08233-0 10213546

[B19] Rockhill B Colditz GA Rosner B Bias in breast cancer analyses due to error in age at menopause Am J Epidemiol 2000 151 404 408 10695599 10.1093/oxfordjournals.aje.a010220

[B20] Ahlgren M Melbye M Wohlfahrt J Sorensen TI Growth patterns and the risk of breast cancer in women N Engl J Med 2004 351 1619 1626 10.1056/NEJMoa040576 15483280

[B21] Ewertz M Duffy SW Adami HO Kvale G Lund E Meirik O Mellemgaard A Soini I Tulinius H Age at first birth, parity and risk of breast cancer: a meta-analysis of 8 studies from the Nordic countries Int J Cancer 1990 46 597 603 10.1002/ijc.2910460408 2145231

[B22] McDowell MA Brody DJ Hughes JP Has age at menarche changed? Results from the National Health and Nutrition Examination Survey (NHANES) 1999–2004 J Adolesc Health 2007 40 227 231 10.1016/j.jadohealth.2006.10.002 17321422

[B23] Nichols HB Trentham-Dietz A Hampton JM Titus-Ernstoff L Egan KM Willett WC Newcomb PA From menarche to menopause: trends among US Women born from 1912 to 1969 Am J Epidemiol 2006 164 1003 1011 10.1093/aje/kwj282 16928728

[B24] Matthews TJ Hamilton BE Mean age of mother, 1970–2000 Natl Vital Stat Rep 2002 51 1 1 16 12564162

[B25] de Waard F Thijssen JH Hormonal aspects in the causation of human breast cancer: epidemiological hypotheses reviewed, with special reference to nutritional status and first pregnancy J Steroid Biochem Mol Biol 2005 97 451 458 10.1016/j.jsbmb.2005.08.005 16230007

[B26] Kaplowitz PB Link between body fat and the timing of puberty Pediatrics 2008 121 Suppl 3 S208 S217 10.1542/peds.2007-1813F 18245513

[B27] Ortiz AP Harlow SD Sowers M Nan B Romaguera J Age at natural menopause and factors associated with menopause state among Puerto Rican women aged 40–59 years, living in Puerto Rico Menopause 2006 13 116 124 10.1097/01.gme.0000191207.28362.22 16607107

[B28] Hardy R Mishra GD Kuh D Body mass index trajectories and age at menopause in a British birth cohort Maturitas 2008 59 4 304 314 18406083 10.1016/j.maturitas.2008.02.009 PMC3500688

[B29] Gail MH Brinton LA Byar DP Corle DK Green SB Schairer C Mulvihill JJ Projecting individualized probabilities of developing breast cancer for white females who are being examined annually J Natl Cancer Inst 1989 81 1879 1886 10.1093/jnci/81.24.1879 2593165

[B30] Chen J Pee D Ayyagari R Graubard B Schairer C Byrne C Benichou J Gail MH Projecting absolute invasive breast cancer risk in white women with a model that includes mammographic density J Natl Cancer Inst 2006 98 1215 1226 16954474 10.1093/jnci/djj332

[B31] Madigan MP Ziegler RG Benichou J Byrne C Hoover RN Proportion of breast cancer cases in the United States explained by well-established risk factors J Natl Cancer Inst 1995 87 1681 1685 10.1093/jnci/87.22.1681 7473816

[B32] Hunter DJ Riboli E Haiman CA Albanes D Altshuler D Chanock SJ Haynes RB Henderson BE Kaaks R Stram DO A candidate gene approach to searching for low-penetrance breast and prostate cancer genes Nat Rev Cancer 2005 5 977 985 10.1038/nrc1754 16341085

[B33] Pharoah PD Tyrer J Dunning AM Easton DF Ponder BA Association between common variation in 120 candidate genes and breast cancer risk PLoS Genet 2007 3 e42 17367212 10.1371/journal.pgen.0030042 PMC1828694

[B34] Hunter DJ Kraft P Jacobs KB Cox DG Yeager M Hankinson SE Wacholder S Wang Z Welch R Hutchinson A A genome-wide association study identifies alleles in FGFR2 associated with risk of sporadic postmenopausal breast cancer Nat Genet 2007 39 870 874 10.1038/ng2075 17529973 PMC3493132

[B35] Cox A Dunning AM Garcia-Closas M Balasubramanian S Reed MW Pooley KA Scollen S Baynes C Ponder BA Chanock S A common coding variant in CASP8 is associated with breast cancer risk Nat Genet 2007 39 352 358 10.1038/ng1981 17293864

[B36] Gail MH Anderson WF Garcia-Closas M Sherman ME Absolute risk models for subtypes of breast cancer J Natl Cancer Inst 2007 99 1657 1659 10.1093/jnci/djm228 18000214

[B37] Hunter DJ Khoury MJ Drazen JM Letting the genome out of the bottle – will we get our wish? N Engl J Med 2008 358 105 107 10.1056/NEJMp0708162 18184955

[B38] Haga SB Willard HF Audeh MW Hunter DJ Khoury MJ Drazen JM Letting the genome out of the bottle N Engl J Med 2008 358 2184 2185 10.1056/NEJMc086053 18480218

[B39] Monninkhof EM Elias SG Vlems FA van dT I Schuit AJ Voskuil DW van Leeuwen FE Physical activity and breast cancer: a systematic review Epidemiology 2007 18 137 157 10.1097/01.ede.0000251167.75581.98 17130685

[B40] Smith-Warner SA Spiegelman D Yaun SS Brandt PA van den Folsom AR Goldbohm RA Graham S Holmberg L Howe GR Marshall JR Alcohol and breast cancer in women: a pooled analysis of cohort studies JAMA 1998 279 535 540 10.1001/jama.279.7.535 9480365

[B41] Breast cancer and hormone replacement therapy: collaborative reanalysis of data from 51 epidemiological studies of 52,705 women with breast cancer and 108,411 women without breast cancer. Collaborative Group on Hormonal Factors in Breast Cancer Lancet 1997 350 1047 1059 Erratum in: Lancet 1997 15;350(9089):1484. 10.1016/S0140-6736(97)08233-0 10213546

[B42] U.S. Census Bureau http://www.census.gov

